# Association of school leaders’ COVID-19 health literacy with the implementation of health promotion in schools in Germany: a cross-sectional study

**DOI:** 10.1186/s12889-025-24196-9

**Published:** 2025-08-21

**Authors:** Marlene Meyer, Kevin Dadaczynski, Melanie Messer, Orkan Okan

**Affiliations:** 1 WHO Collaborating Centre for Health Literacy, TUM Health Literacy Unit, Department of Health and Sport Sciences, TUM School of Medicine and Health,, Technical University of Munich, Munich, Germany; 2 Department Sport and Health Sciences, University of Potsdam, Potsdam, Germany; 3 Centre for Applied Health Science, Leuphana University Lueneburg, Lueneburg, Germany; 4 Institute of Nursing Science, University Hospital Würzburg, Würzburg, Germany; 5 Department of Nursing Science, University of Würzburg, Würzburg, Germany

**Keywords:** COVID-19, Health literacy, Health promoting school, Multiple linear regression analysis

## Abstract

**Background:**

Health literacy has recently been proposed as a resource to deal with health-related information during the COVID-19 pandemic. Especially the education sector was affected by the consequences of the pandemic, e.g. through school closures and reopenings by following strict hygiene regulations. During challenging times like these, school principals are the key actors in the school environment. They are not only responsible for school functioning, but school principals’ behavior and attitudes are also associated with health promoting school activities. The present study aimed to assess COVID-19 health literacy levels of German school principals, and to investigate the associations of COVID-19 health literacy with the implementation of health promotion in schools during the pandemic.

**Methods:**

As part of the joined studies coordinated by the COVID-HL Network, the COVID-19 Health Literacy School Principal Survey was conducted in Germany from March to April 2021. 2187 school principals and school management team members from four German federal states participated in the online survey. The HLS-COVID-Q22 was used to assess self-reported COVID-19 health literacy. The COVID-19 related HPS implementation scale was used to measure a holistic approach to school health promotion, consisting of 3 subscales: COVID-19 related support for students, staff and school. COVID-19 health literacy levels were computed using Rasch analysis. Multiple linear regression analysis was conducted to examine the association of school principals’ self-reported COVID-19 health literacy and the implementation of COVID-19 related health promotion in schools.

**Results:**

School principals showed good self-reported COVID-19 health literacy. Multiple linear regression analysis revealed that self-reported COVID-19 health literacy positively predicted the implementation of COVID-19 related health promotion in schools for all three subscales during the pandemic. In some regression models, the sociodemographic factors age, sex, and school type were also significant factors.

**Conclusions:**

German school principals had high self-reported COVID-19 health literacy. School principals’ self-reported COVID-19 health literacy was a significant factor associated with the implementation of COVID-19 related health promotion in schools. The present findings underline the need to investigate the relationship of school principals’ health literacy and other potential influencing factors for the implementation of health promotion in schools.

**Supplementary Information:**

The online version contains supplementary material available at 10.1186/s12889-025-24196-9.

## Background

The COVID-19 pandemic has been accompanied by an increase in health information regarding the pandemic [1, 2]. Through different communication technologies, health information were rapidly spread, distributing correct information as well as mis- and disinformation at a fast-moving rate. This led to an “infodemic”, which has been classified as a threat to public health by the World Health Organization [3, 4]. A common problem is the mere amount of health information, as the discrimination of reliable and unreliable information becomes increasingly difficult [5]. A resource to manage health information during the COVID-19 pandemic is health literacy [6, 7]. Health literacy (HL) can be defined as the ability to access, understand, appraise and apply health information to reach health-related decisions [8]. Accordingly, COVID-19 HL can be defined as the ability to access, understand, appraise and apply health information to reach health-related decisions in the context of the COVID-19 pandemic. For instance, people with lower COVID-19 HL were more likely to be confused by information regarding the COVID-19 pandemic [9]. In general, differences in HL levels were associated with different health outcomes like increased hospitalizations or greater emergency care use [10].

The HLS-COVID-Q22 questionnaire has been developed to assess self-reported COVID-19 HL [9]. For self-report questionnaires, different methods to compute HL levels exist. Most commonly, mean scores or sum scores are calculated and people are classified into ability levels based on specific cut-off values [9, 11, 12]. For the HLS-COVID-Q22, it is proposed to classify people with a mean score of ≤ 2.5 as having “inadequate health literacy”, a mean score of 2.5 < mean score < 3 as having “problematic health literacy” and a mean score of ≥ 3 as having “sufficient health literacy” [9]. While this classification technique of HL levels is based on the widely used HLS-EU-Q47 questionnaire concept [9, 13], it has been criticized to adopt cut-off values from a previous questionnaire to a new questionnaire [14]. Setting the same cut-off values (e.g., achieved percentage of the sum/mean score) for different questionnaires is flawed, because the difficulty of the test and the difficulty of the items will be disregarded [14, 15]. Additionally, dividing people into different competency levels should be based on differences in competence and not based on existing cut-off values from other questionnaires.

The COVID-19 pandemic has been a challenge for almost every area of life. Especially the education sector was negatively impacted through school closures, the change from face-to-face to online teaching as well as the implementation of hygiene regulations [16–18]. In a systematic review, adverse effects on mental health, teaching and learning, quality of life, and physical health were identified in school populations, i.e. in students, teachers, parents, and school administration [16]. Particularly, children and adolescents were affected by the consequences of the pandemic. The pandemic led to deficits in the learning progress of children and adolescents, mostly in children from disadvantaged socio-economic backgrounds, thereby intensifying socio-demographic inequalities [19]. In addition, short-term and long-term pandemic-associated adverse effects on mental health and health outcomes in school-aged children and adolescents are of concern in public health [20, 21].

Similarly, school staff was negatively affected by the consequences of the pandemic [16]. Increases in teachers’ levels of anxiety, depression and stress have been found, probably due to increased job demands, the transition to online teaching and a disturbed work-life balance [22, 23]. Dadaczynski et al. emphasize that especially school principals might be exposed to an exceptionally high level of stress as they held responsibility for the entire school [24]. Previous studies have indicated that school principals represent a professional group that is mentally and physically affected by work-related demands [25, 26]. Since school principals behavior and attitudes (e.g., instructional management, internal relations, emotional support) have been linked to student achievement, teacher well-being as well as health promoting school activities [27, 28], it is critical to investigate how school principals dealt with the pandemic.

The influence of childhood experiences is vital for the health status in adult life [29, 30]. Schools are considered an essential setting for health promotion, since children usually spend a significant amount of time in school [31]. There are numerous health-promoting programs and interventions in schools, targeting specific topics and audiences [32–34]. However, school-based health interventions tend to lack sustainability after the end of funding or external implementation support [35, 36]. The Health Promoting School (HPS) framework is a holistic approach that goes beyond the individual and addresses the whole school community [37]. The WHO defined six components of a HPS: healthy school policies, healthy physical school environments, healthy school social environments, health skills and education, links with parents and the school community and access to school health services [38, 39]. While different definitions of HPS exist, HPS mainly aims to facilitate health through health education, ethos and environment of the school, as well as engagement with families and communities [40]. In a systematic review, the HPS approach was found to be effective in enhancing student health [31]. Committed school principals and leadership support are proposed to be central to implementing and sustaining HPS in schools [28, 35, 36].

Preliminary results in Germany have already shown that school principals experienced increased stress due to the pandemic through additional job demands, resulting in maladaptive coping strategies such as working extra hours in their free time or sacrificing leisure activities in favor of work [41, 42]. HL might not only be a personal resource for school principals’ own health, but also an influencing factor for HPS implementation. A recent study showed that male school principals’ health literacy levels were associated with the implementation of health promoting school activities [43]. Evidence on school principals’ characteristics on HPS implementation is scarce [28]. Recently however, associations between school principals’ sociodemographic characteristics and HPS implementation were found [44]. The aim of the present study is to determine school principals’ COVID-19 HL levels as a resource in managing health information about the pandemic, and to investigate the influence of COVID-19 HL as well as school principals’ sociodemographic characteristics on the implementation of COVID-19 related health promotion in schools in Germany. Additionally, a different method to compute HL levels for self-report questionnaires will be proposed.

## Methods

### Study design and study population

The COVID-19 Health Literacy Network (COVID-HL Network; now renamed the Global Health Literacy Research Network, GLOBHL, www.globhl.org) was launched at the beginning of 2020 [41]. The network aims to advance global health literacy research for diverse populations and settings to generate insights that inform public health policy and practice. In light of the critical role of school principals and members of the school management team (e.g., deputy principals), the COVID-19 Health Literacy School Principal Survey (COVID-HL school survey) was launched by the network in 11 countries (Germany, Denmark, Italy, Poland, Taiwan, United Kingdom, China, Greece, Romania, Switzerland, Turkey) to asses school principals’ health literacy in the context of the pandemic [45, 46]. Data collection in Germany was from March 9 to April 13 2021, hence, during the third wave of the pandemic in four out of 16 German federal states (Baden-Württemberg, Hesse, Lower Saxony, and North Rhine-Westphalia) [41, 47]. School principals, deputy school principals and members of the school management team took part in the survey. In cooperation with the school principals’ associations of the four federal states, school principals received an invitation to participate in the study through e-mail communication by the respective association. To increase the participation rate, a second e-mail was send a couple of days later. Participants gave written informed consent before granting access to the online study. The study was approved by the Bielefeld University Ethics Board (Reference No 2021-030) and the educational ministry of Baden-Württemberg, Hesse and Lower Saxony.

The characteristics of study participants are displayed in Table 1. *N* = 2187 participants completed the online questionnaire. The majority of participants were female principals, deputy principals, or members of the school management team (66.1% = female, 33.8% = male, 0.1% = did not specify) [41, 47]. The mean age was *M* = 51 years (range = 27–68 years). School principals mainly completed the questionnaire themselves (84.5%). Of the sample, 15.5% were school principal deputies or members of the school management team. Around half worked at a primary school (53.2%) and the other half at a secondary school (46.8%).

**Table 1:** Characteristics of study participants (*n* = 2187)

Item	Category	Percentage (%)	Frequency
Sex	Male	33.8	739
	Female	66.1	1446
	No specification	0.1	1
Age	≤ 45 years	21.8	476
	46–55 years	46.2	1008
	≥ 56 years	32.0	699
Position	School principal	84.5	1833
	Deputy	15.5	336
School type	Primary school	53.2	1138
	Secondary school	46.8	1003

Note. Some responses were missing for some demographic variables

### Measurement tools

The COVID-HL school survey consisted of several demographic variables and health-related constructs [41]. Self-reported COVID-19 HL was assessed through the HLS-COVID-Q22 questionnaire. The development and validation of the HLS-COVID-Q22 has been described elsewhere [9]. A psychometric evaluation study of the current sample using Rasch analysis can be found at Meyer et al. [47]. It consists of the 4 subscales “accessing” (6 items), “understanding” (6 items), “appraising” (5 items) and “applying” (5 items) health-related information in the context of the COVID-19 pandemic. In sum, there are 22 items. Participants were asked how easy or difficult they perceive the items on a 4-point likert-type rating scale (“Very difficult”, “Difficult”, “Easy”, “Very easy”).

COVID-19 related implementation of health promotion in schools (COVID-19 related HPS implementation) was assessed through a self-developed scale based on previous work [44, 48]. Detailed description of the scale and its validation for the current sample can be found at Dadaczynski et al. [45]. The COVID-19 related HPS implementation scale was developed to capture the three core dimensions of a holistic approach to school health promotion and consists of 3 subscales: COVID-19 related support for students (HPS-students; α = 0.76), health-promoting teaching, learning and working conditions (HPS-staff; α = 0.81) and principles of the health promoting school (HPS-school; α = 0.65). Participants were asked on a 4-point likert-type rating scale (“Not true at all”, “Mostly not true”, “Likely to be true”, “Totally true”) to which extent activities on COVID-19 related HPS are implemented at their school. Item 2 is an example for the subscale HPS-students: “At our school, students learn ways to protect themselves from infection.”, Item 6 for HPS-staff: “At our school, school staff are supported in dealing with stressful situations caused by the coronavirus (e.g. stress).” and Item 11 for HPS-school: “At our school, we work closely with parents when it comes to promoting and protecting children’s health.” [45]. The scale originally consisted of 15 items. Based on the PCA analyses and recommendation of Dadaczynski at al., three items were excluded for data analysis. Supplementary Table 1 displays the item descriptions.

### Data analyses

#### Data exclusion

In summary, *n* = 156 participants were excluded from data analysis. The reasons for exclusion were: not the target population (*n* = 6 participants), the HLS-COVID-Q22 questionnaire has not been answered (*n* = 83 participants), and the maximal possible raw score on the HLS-COVID-Q22 was achieved (*n* = 67 participants). Participants with the maximal possible raw score on the HLS-COVID-Q22 are termed as “extreme participants”. Here, the real participant’s ability is not measurable, because the real participant’s ability could be exactly at the maximal possible raw score of the questionnaire or in an unquantifiable amount above [14]. This results in an infinite measurement error, which is the reason why, “extreme participants” should be excluded from data analysis. Accordingly, participants with the minimal possible raw score on the questionnaire would have been excluded if there had been any in the data set. Therefore, 2031 participants were included in the final data analysis to determine the COVID-19 HL levels. For the regression analysis, participants were additionally excluded who did not answer the COVID-19 related HPS implementation subscales (*n* = 34–38 participants) and who achieved the maximal possible raw score (*n* = 64–183 participants) or the minimal possible raw score (*n* = 2–21 participants) on the COVID-19 related HPS implementation subscales.

#### Covid-19 health literacy levels

For data analysis, IBM SPSS version 29.0 was used. Descriptive statistics were calculated (*M*, SD, %). Univariate analyses for sociodemographic differences in COVID-19 HL and COVID-19 related HPS implementation were computed using t-tests. To calculate the self-reported COVID-19 HL levels of school principals, data preprocessing needed to be conducted since the HLS-COVID-Q22 is a likert-type rating scale. Because of the categorical nature of the items, Rasch analysis was administered to transform the responses into linear measures [14]. The data set was analyzed with WINSTEPS^®^ software, using the Partial Credit Model for polytomous data [15, 49]. The responses of a participant to the items of the HLS-COVID-Q22 were calculated into an interval scaled linear person measure that reflects this participant’s ability (self-reported COVID-19 HL) [14]. The results will be displayed in a Wright-like map in which the person measures are plotted by number of participants. Participants with higher person measures were more agreeable to the items (i.e. indicating higher self-reported COVID-19 HL), while participants with lower person measures were less agreeable to the items (i.e. indicating lower self-reported COVID-19 HL). Applying Rasch analysis, reliable person measures can be computed with missing responses to items. In the present survey, there were 1.3% of missing data.

There are different ways to compute HL levels based on the type of questionnaire. Typically, mean scores or sum scores are calculated [9, 11, 12]. Then, participants are sorted into four or three ability levels based on specific cut-off values. For the HLS-COVID-Q22, Orkan et al. proposed a mean score of ≤ 2.5 as “inadequate health literacy”, a mean score of 2.5 < mean < 3 as “problematic health literacy” and a mean score of ≥ 3 as “sufficient health literacy” [9]. When translated into percentages a score of ≤ 50% would fall into “inadequate health literacy”, a score of 50% < score < 66% into “problematic health literacy” and a score of ≥ 66% into “sufficient health literacy”. For comparison purposes with previous studies, COVID-19 HL levels based on those cut-off values will be reported.

A second approach of categorizing participants into ability levels will be reported using Rasch analysis. Rasch-Thurstonian thresholds were computed for each item for each rating scale category, i.e. the 50% cummulative probability threshold between each pair of adjoining categories (“Very difficult”/“Difficult”, “Difficult”/“Easy”, “Easy”/“Very easy”) [14, 15]. If a participant has a person measure that is the cut-off value between two categories (e.g. “Difficult”, “Easy”), that participant has a probability of 50% performing according to the lower categories (choosing “Difficult” or lower) and a 50% probability of performing according to the higher categories (choosing “Easy” or higher). Then a median value was computed from all Rasch-Thurstonian threshold values of the 22 items to determine the three cut-off values between the four categories.

#### Regression analysis

To investigate the association of self-reported COVID-19 HL on COVID-19 related HPS implementation in German schools, multiple linear regression analysis has been conducted. Prior to the regression analysis, Pearson correlation was conducted to assess the associations between the outcome and explanatory variables. Effect sizes were classified by convention by Cohen (low: *r* =.1, moderate: *r* =.3, high: *r* =.5) [50]. To gain a nuanced insight into the association, three blockwise multiple linear regression analyses were conducted. The outcome variable was the respective subscale of COVID-19 related HPS implementation (students, staff, school). In Block 1, the regression models were controlled for sociodemographic characteristics of the school principals, i.e., age (metric variable), sex (male vs. female) and school type (primary school vs. secondary school). In Block 2, the explanatory variable COVID-19 HL was administered. When conducting a multiple linear regression analysis, the outcome variable needs to be metric or ‘metric defined’ (at least 5 values that are thought to be interval scaled, e.g. 5-point likert-type rating) [51]. Since the COVID-19 related HPS implementation subscales are 4-point likert-type rating scales and have categorical items, data preprocessing needed to be conducted. For every subscale, a Rasch analysis was administered to transform the responses into linear measures [14]. The data set was analyzed with WINSTEPS^®^ software, using the Andrich’s Rating Scale Model for polytomous data [15, 52]. Then, the interval scaled linear person measures were used as outcome variable. The explanatory variables in a multiple linear regression analysis should be metric, ‘metric defined’ or binary [51]. All explanatory variables met the requirements.

Then, the model assumptions of a multiple regression analysis were verified [53]. Multicollinearity was examined using Pearson correlation and computing the variance inflation factor (VIF) and tolerance of the explanatory variables. Correlation of >|0.80| between two explanatory variables and VIF > 10 or tolerance < 0.1 of one explanatory variable might indicate multicollinearity [54]. Normal distribution of residuals was examined visually inspecting the residuals-histogram, because of the large sample size. Homoscedasticity was examined visually via scatterplot of the residuals against the fitted values of the regression model and analytically using the Breusch-Pagan test. The estimated fit of the regression model was provided by R^2^. P-values < 0.05 were considered statistically significant.

For all three regression models, multicollinearity was not found. There were no Pearson correlations of two explanatory variables higher than|0.80|. In addition, no explanatory variable showed a VIF value > 10 or a tolerance value < 0.1. Normal distribution of residuals could be assumed based on the residuals-histograms. The scatterplots of the residuals against the fitted values of the regression model showed no remarkable changes in variance of the residuals. Additionally, the Breusch-Pagan tests were not significant. Homoscedasticity was assumed.

## Results

### Descriptive and univariate analyses

Table 2. shows the frequencies of every response category of every item of the HLS-COVID-Q22. Participants chose the category “Easy” the most, with a mean of 50.25% over all items. “Very easy” was chosen on average by 32.25%, “Difficult” by 15.7% and “Very difficult” by 1.8%. Descriptively speaking, participants were agreeable to the items.

**Table 2.** Frequencies in percent of the HLS-COVID-Q22 items.

Item	On a scale from very easy to very difficult, how easy would you say it is to…	Very difficult	Difficult	Easy	Very easy
1	…find information about the coronavirus on the internet?	0.2	3.0	40.2	56.6
2	…find information on the internet about protective behaviours that can help to prevent infection with the coronavirus?	0.4	4.9	43.1	51.6
3	…find information in newspapers, magazines and on TV about behaviours that can help to prevent infection with the coronavirus?	1.1	10.2	48.9	39.8
4	…find out information how to recognize if I am likely to be infected with the coronavirus?	0.9	10.4	48.9	39.8
5	…find information on how to find professional help in case of a coronavirus infection?	1.1	14.5	47.6	36.7
6	…find information on how much I am at risk for infection with the coronavirus?	3.4	28.6	46.4	21.6
7	…understand your doctor’s, pharmacist’s or nurse’s instructions on protective measures against a coronavirus infection?	0.3	2.9	50.6	46.1
8	…understand recommendations of authorities regarding protective measures against a coronavirus infection?	5.8	34.2	42.7	17.2
9	…understand advice from family members or friends regarding protective measures against a coronavirus infection?	0.8	10.2	57.2	31.8
10	…understand information in the media on how to protect myself against a coronavirus infection?	0.5	7.2	53.8	38.5
11	…understand risks of the coronavirus that I find on the internet?	0.6	11.2	53.7	34.5
12	…understand risks of the coronavirus that I find in newspapers, magazines or on TV?	0.6	11.3	54.5	33.7
13	…judge if information on the coronavirus and the coronavirus epidemic in the media is reliable?	9.7	38.4	40.2	11.7
14	…judge which behaviours are associated with a higher risk of a coronavirus infection?	1.6	12.9	52.5	33.1
15	…judge what protective measures you can apply to prevent a coronavirus infection?	0.6	9.5	52.8	37.0
16	…judge how much I am at risk for a coronavirus infection?	3.2	32.8	44.3	19.6
17	…judge if I have been infected with the coronavirus?	3.5	41.1	44.3	11.1
18	…decide how you can protect yourself from a coronavirus infection based on information in the media?	1.0	12.3	59.2	27.5
19	…follow instructions from your doctor or pharmacist regarding how to handle the coronavirus situation?	0.3	5.8	58.5	35.3
20	…use information the doctor gives you to decide how to handle an infection with the coronavirus?	0.5	8.6	58.9	32.1
21	…use media information to decide how to handle an infection with coronavirus?	2.1	23.6	53.6	20.7
22	…to behave in a way to avoid infecting others?	1.4	11.6	53.5	33.6

Note. The German version of the HLS-COVID-Q22 was used in the study. For reporting purposes, the English version of the items is displayed

In Table 3, mean values and standard deviations of COVID-19 HL and the three subscales of COVID-19 related HPS implementation (students, staff, school) are stratified by sociodemographic characteristics. School principals from secondary schools showed significantly higher self-reported COVID-19 HL, *t*(1984) = −4.87, *p* <.05, *d* = −0.22, whereas no sex or age differences were found. Principals from primary schools reported significantly higher COVID-19 related HPS implementation for the subscale students, *t*(1768) = 2.80, *p* <.05, *d* = 0.13, and the subscale school, *t*(1885) = 4.06, *p* <.05, *d* = 0.19). Female school principals reported significantly higher COVID-19 related HPS implementation for the subscale students, *t*(1807) = −3.23, *p* <.05, *d* = −0.16, and the subscale school, *t*(1924) = −4.41, *p* <.05, *d* = −0.21). Older school principals reported significantly higher COVID-19 related HPS implementation for the subscale staff, *t*(1828) = −2.56, *p* <.05, *d* = −0.12.

**Table 3:** COVID-19 HL and COVID-19 related HPS implementation stratified by sociodemographic characteristics

Category	Mean (SD)				
	COVID-19 HL (*n* = 2031)	HPS-students (*n* = 1811)		HPS-staff (*n* = 1830)	HPS-school (*n* = 1926)
Sex		*			*
Male	2.07 (1.83)	2.32 (1.83)		1.15 (3.26)	0.92 (1.68)
Female	2.08 (1.87)	2.61 (1.79)		1.28 (3.09)	1.28 (1.65)
Age				*	
≤ 50 years	2.13 (1.85)	2.47 (1.83)		1.03 (3.21)	1.11 (1.68)
≥ 51 years	2.03 (1.86)	2.55 (1.79)		1.42 (3.09)	1.20 (1.66)
School type	*	*			*
Primary school	1.88 (1.83)	2.62 (1.74)		1.38 (3.11)	1.31 (1.61)
Secondary school	2.29 (1.86)	2.34 (1.87)		1.12 (3.12)	1.00 (1.73)

Note. Some responses were missing for some demographic variables

* *p* <.05 (two-tailed tests)

### COVID-19 health literacy levels

**Fig. 1:**
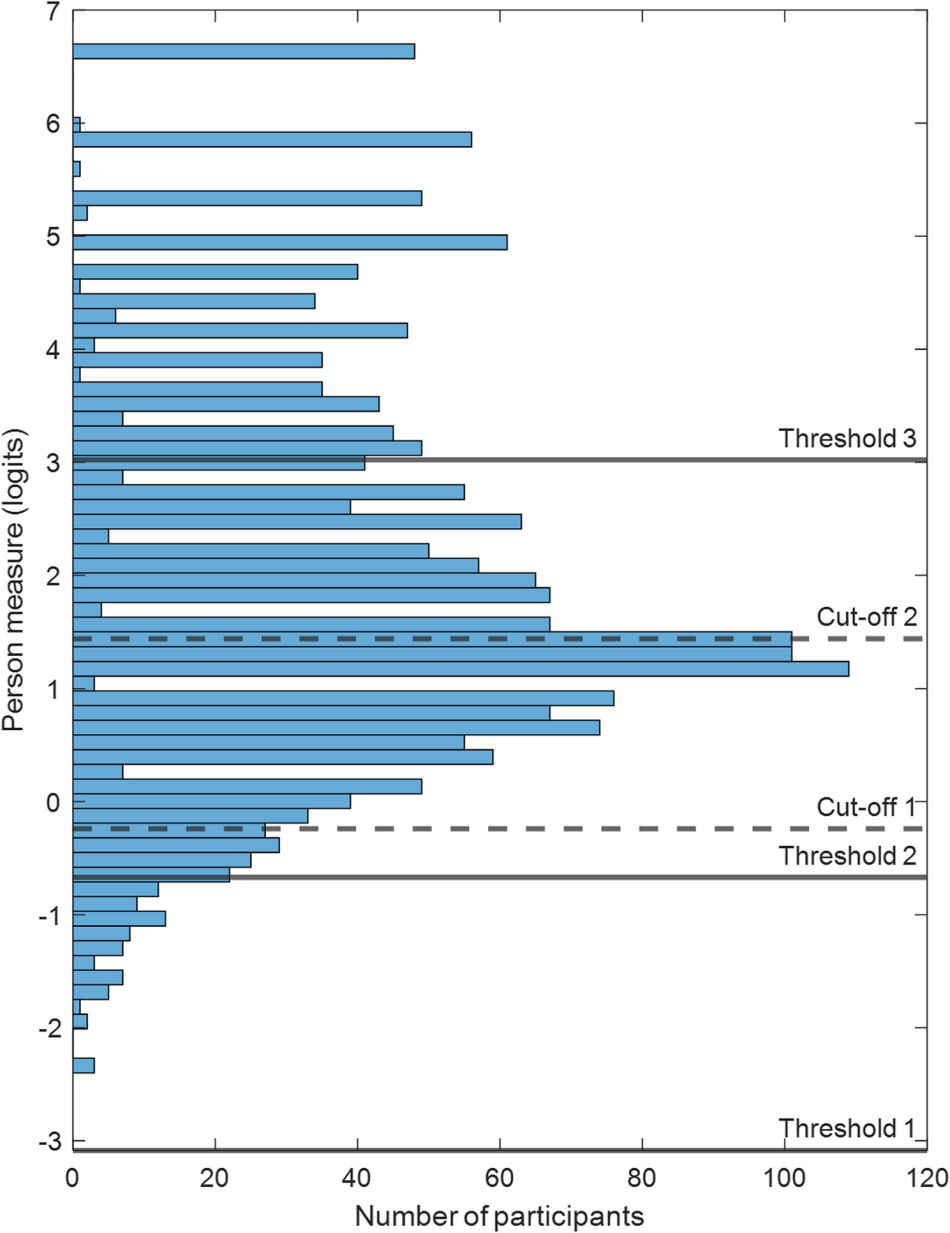
Wright-like map. Person measures are plotted by number of participants. Higher person measures indicate people with higher self-reported COVID-19 HL, while lower person measures indicate people with lower self-reported COVID-19 HL. The COVID-19 HL level boundaries are shown for the common technique (Cut-off 1 = 50% and Cut-off 2 = 66%) and the proposed Rasch-Thurstonian threshold method (Threshold 1, 2 & 3)

The person measures are displayed by number of participants in Fig. 1. The computed cut-off values for both competency level computation techniques are drawn into the plot. Person measures range from − 2.37 to 6.68 logits and the mean person measure is 2.07 logits. Using the common technique to compute three COVID-19 HL levels, the first cut-off needs to be at 50% of the maximal possible raw score and the second cut-off at 66% [9]. The HLS-COVID-Q22 has a maximal possible raw score of 88 and a minimal possible raw score of 22 (22 items with 4 response categories). 50% is at the raw score of 55 (88–22 = 66; 50% of 66 = 33; 22 + 33 = 55) and 66% at the raw score of rounded up to 66 (66% of 66 = 43.56; 22 + 43.56 = 65.56). Supplementary Table 2 shows the estimated person measures for every possible raw score. A raw score of 55 translates to a person measure of − 0.24 logits, defining the value of Cut-off 1 (between “inadequate” and “problematic” COVID-19 HL). A raw score of 66 translates to a person measure of 1.44, defining the value of Cut-off 2 (between “problematic” and “sufficient” COVID-19 HL). This resulted in 7.3% of school principals showing a self-reported “inadequate”, 34.3% a “problematic” and 58.4% a “sufficient” COVID-19 HL level.

Using Rasch-Thurstonian thresholds, Threshold 1 (between “Very difficult” and “Diffult”) is at −3.085 logits. Threshold 2 (between “Difficult” and “Easy”) is at −0.67 logits. Threshold 3 (between “Easy” and “Very easy”) is at 3.02 logits. This resulted in 0% of school principals falling into Level 1, 3.4% into Level 2, 68.6% into Level 3 and 28% into Level 4 of self-reported COVID-19 HL.

### Regression analysis

Pearson correlations for all three multiple linear regression analyses are shown in Table 4. For the first subscale, the explanatory variables COVID-19 HL (*r* =.25) and sex (*r* =.08) were significantly positively associated with the outcome variable COVID-19 related HPS implementation (students), while school type (*r* = -.07) was negatively associated. The explanatory variables showed no correlation above|*r*| = 0.38 with each other. For the second subscale, the explanatory variables COVID-19 HL (*r* =.16) and age (*r* =.08) were significantly associated with the outcome variable COVID-19 related HPS implementation (staff). In addition, the explanatory variables showed low to moderate correlations with each other (|r| = 0.01 to 0.38). For the third subscale, the explanatory variables COVID-19 HL (*r* =.16) and sex (*r* =.10) were significantly positively associated with the outcome variable COVID-19 related HPS implementation (school), while school type (*r* = -.09) was negatively associated. The explanatory variables showed no correlation above|*r*| = 0.38 with each other.

**Table 4:** Pearson correlations of explanatory and outcome variables

	1	2	3	4	5
1 HPS-students	-				
2 Age	0.05	-			
3 Sex	0.08*	−0.01	-		
4 School type	−0.07*	0.11*	−0.38*	-	
5 COVID-19 HL	0.25*	−0.03	0.00	0.11*	-
1 HPS-staff	-				
2 Age	0.08*	-			
3 Sex	0.02	−0.02	-		
4 School type	−0.04	0.11*	−0.38*	-	
5 COVID-19 HL	0.16*	−0.01	0.01	0.11*	-
1 HPS-school	-				
2 Age	0.03	-			
3 Sex	0.10*	−0.004	-		
4 School type	−0.09*	0.10*	−0.38*	-	
5 COVID-19 HL	0.16*	−0.02	0.002	0.10*	-

Note. *HPS* = COVID-19 related health promotion in school, *HL* = health literacy

* *p* <.05, two-tailed tests

Table 5 displays the results of the blockwise multiple linear regression analysis with the subscale COVID-19 related HPS implementation regarding students. In Block 1, all explanatory variables (age, sex, school type) have shown to be significant factors associated with COVID-19 related HPS implementation. After adding Block 2, only age (*B* = 0.014, *p* =.009) and school type (*B* = − 0.306, *p* <.001) remain significant factors. Additionally, self-reported COVID-19 HL (*B* = 0.252, *p* <.001) revealed to be a significant factor. The overall regression model was statistically significant, explaining 7.1% of variance of COVID-19 related HPS implementation regarding students by the explanatory variables (adjusted *R*^2^ = 0.071, *F*(4, 1760) = 34.598, *p* <.001).

**Table 5:** Multiple linear regression analysis. Outcome: COVID-19 related HPS implementation-students

	B	SE	β	*p*	Tolerance	VIF	*R* ^2^	adj. *R*^2^	∆*R*^2^	*p*
Block 1							0.010	0.008		
Constant	1.834	0.302		< 0.001						
Age	0.012	0.006	0.051	0.034	0.987	1.014				
Sex	0.214	0.097	0.056	0.028	0.857	1.267				
School type	− 0.185	0.093	− 0.051	0.047	0.846	1.182				
Block 2							0.073	0.071	0.063	< 0.001
Constant	1.302	0.296		< 0.001						
Age	0.014	0.006	0.061	0.009	0.985	1.015				
Sex	0.164	0.094	0.043	0.082	0.855	1.170				
School type	− 0.306	0.091	− 0.085	< 0.001	0.834	1.200				
COVID-19 HL	0.252	0.023	0.253	< 0.001	0.984	1.016				

Note. *HPS* = health promotion in school, *HL* = health literacy, *school type* = primary school vs. secondary school

The results of the blockwise multiple linear regression analysis with the subscale COVID-19 related HPS implementation regarding staff are displayed in Table 6. In Block 1, age was a significant factor. In Block 2, age (*B* = 0.039, *p* <.001), school type (*B* = − 0.465, *p* =.004) and self-reported COVID-19 HL (*B* = 0.287, *p* <.001) revealed to be significant factors associated with COVID-19 related HPS implementation. The overall regression model was statistically significant, explaining 3.5% of variance of COVID-19 related HPS implementation regarding staff by the explanatory variables (adjusted *R*^2^ = 0.035, *F*(4, 1782) = 17.216, *p* <.001).

**Table 6:** Multiple linear regression analysis. Outcome: COVID-19 related HPS implementation-staff

	B	SE	β	*p*	Tolerance	VIF	*R* ^2^	adj. *R*^2^	∆*R*^2^	*p*
Block 1							0.010	0.008		
Constant	− 0.549	0.530		0.301						
Age	0.038	0.010	0.090	< 0.001	0.988	1.012				
Sex	0.024	0.171	0.004	0.889	0.852	1.174				
School type	− 0.314	0.162	− 0.050	0.053	0.842	1.187				
Block 2							0.037	0.035	0.028	< 0.001
Constant	−1.083	0.528		0.040						
Age	0.039	0.010	0.093	< 0.001	0.987	1.013				
Sex	− 0.051	0.169	− 0.008	0.763	0.849	1.178				
School type	− 0.465	0.161	− 0.074	0.004	0.828	1.208				
COVID-19 HL	0.287	0.040	0.167	< 0.001	0.983	1.018				

Note.  HPS =health promotion in school, *HL* = health literacy, *school type* = primary school vs. secondary school

Table 7 displays the results of the blockwise multiple linear regression analysis with the subscale COVID-19 related HPS implementation regarding school. In Block 1, sex and school type were significant factors. In Block 2, sex (*B* = 0.209, *p* =.016) and school type (*B* = − 0.311, *p* <.001) remain significant factors. Additionally, self-reported COVID-19 HL (*B* = 0.154, *p* <.001) revealed to be a significant factor associated with COVID-19 related HPS implementation. The overall regression model was statistically significant, explaining 4% of variance of COVID-19 related HPS implementation regarding staff by the explanatory variables (adjusted *R*^2^ = 0.040, *F*(4, 1877) = 20.832, *p* <.001).

**Table 7:** Multiple linear regression analysis. Outcome: COVID-19 related HPS implementation-school

	B	SE	β	*p*	Tolerance	VIF	*R* ^2^	adj. *R*^2^	∆*R*^2^	*p*
Block 1							0.014	0.012		
Constant	0.719	0.272		0.008						
Age	0.008	0.005	0.034	0.136	0.989	1.011				
Sex	0.240	0.088	0.067	0.007	0.854	1.171				
School type	− 0.240	0.084	− 0.071	0.004	0.846	1.183				
Block 2							0.043	0.040	0.028	< 0.001
Constant	0.394	0.272		0.147						
Age	0.009	0.005	0.040	0.079	0.988	1.012				
Sex	0.209	0.087	0.059	0.016	0.852	1.174				
School type	− 0.311	0.083	− 0.093	< 0.001	0.834	1.198				
COVID-19 HL	0.154	0.021	0.170	< 0.001	0.986	1.014				

Note. *HPS* = health promotion in school, *HL* = health literacy, *school type* = primary school vs. secondary school

## Discussion

The present study aimed to gain insight into school principals’ COVID-19 HL and to investigate whether COVID-19 HL was linked to the implementation of COVID-19 related health promotion in schools during the pandemic in Germany. In general, school principals’ self-reported COVID-19 HL level were high. Multiple linear regression analysis indicated a positive association between self-reported COVID-19 HL and the three COVID-19 related HPS implementation subscales.

### COVID-19 health literacy levels

Whereas no sex or age differences were found, school principals from secondary schools showed significantly higher mean values of self-reported COVID-19 HL than school principals from primary schools. These findings are in contrast to the Polish COVID-HL school survey, in which no differences of school type were found [55]. In the Turkish COVID-HL school survey, no differences were found between principals from primary and secondary school, but primary school principals had significantly lower mean values of self-reported COVID-19 HL than principals working in high school [56]. In light of these heterogeneous findings, future research needs to investigate the role of school type regarding self-reported COVID-19 or general HL. If there indeed seem to be differences, exploring the reasons would be vital for developing interventions to strengthen school principals HL.

With the common technique to compute three COVID-19 HL levels [9], 7.3% of school principals showed a self-reported “inadequate”, 34.3% a “problematic” and 58.4% a “sufficient” COVID-19 HL level. These findings are in line with the Polish COVID-HL school survey, in which 7.2% of school principals showed a self-reported “inadequate”, 28.0% a “problematic” and 64.8% a “sufficient” COVID-19 HL level [55]. In the Danish COVID-HL school survey, there were more school leaders with a “sufficient” level of COVID-19 HL with 71.4% [57]. However, in the Hong Kong COVID-HL school survey, only 46.3% had a “sufficient” COVID-19 HL level, while 6.2% of school principals showed a self-reported “inadequate” and 47.5% a “problematic” level [58]. These differences between countries are in line with previous research on general health literacy [11], indicating a need for further investigation on country comparisons.

In comparison with a previous German study on school principals’ general health literacy, the current findings are slightly inferior. In a study from 2018, 5.7% of school principals had a self-reported “inadequate”, 23.5% a “problematic” and 70.8% a “sufficient” HL level [59]. However, in this study, a different measurement tool was used and the study was conducted pre-pandemic, which might explain the different findings. Compared to the general German population, school principals seem to show better general and COVID-19 HL levels. A study of the German population indicated that 7.3% of the participants had “excellent” general HL, 38.4% “sufficient” and 54.3% “limited” with 9.7% “inadequate” [60]. But comparisons should be made cautiously as the authors used another technique to categorize participants into HL levels. In 2020, a study found that 34.9% of the general German population had an “inadequate”, 15.2% a “problematic” and 49.9% a “sufficient” COVID-19 HL level [9]. When comparing school principals findings with the findings of the general adult population in Germany, an explanation of the differences might lie in the sociodemographic characteristics of school principals [59]. They represent an occupational group that is well-educated and has a high income, which are known to be relevant determinants of health literacy [61].

As mentioned, there are currently different ways to compute HL levels for self-report questionnaires. The commonality is that they split participants into 3 or 4 levels, and the cut-off criteria are based on the same percentage values gained from the raw mean or sum score, e.g. 50% and 66% [9]. Boone, Staver and Yale argue that categorizing people into different competency levels based on arbitrary numbers should be avoided [14]. For example, in a school setting, students typically receive the best possible grade on a test when they have achieved more than 90%. The authors argue that setting a cut-off point of 90% for every test is meaningless, because the difficulty of the test and the difficulty of the items implicate student knowledge. The 90% alone don’t tell what a student knows and what a student doesn’t know. However, dividing people into different competency levels should be based on differences in competence.

Transferred to health literacy research, HL levels should be based on differences in HL competence. For instance, in a study to determine patients with low HL, the S-TOFHLA was used, which is a performance-based HL screening tool [62]. Here, participants were categorized into “inadequate”, “marginal”, and “adequate” HL based on an achieved score in relation to the sum score. However, the cut-off numbers have meaning, because people who would be categorized as “inadequate” HL would typically not show the competencies needed to achieve a higher level (e.g. “comprehending more complicated passages such as instructions for a radiographic procedure or educational brochures”). The comparison of performance-based and self-report HL tools is difficult for many reasons, the most prominent is that different theoretical concepts of HL are operationalized [63, 64]. Therefore, the classification of the S-TOFHLA cannot just be transferred to any self-report HL tool, because the underlying HL definition might be different. Furthermore, each test is different with varying difficulties of items and competence levels should not be based on arbitrary numbers [14].

In the absence of a theoretical competence-based classification technique of HL levels for the widely used HLS-EU-Q47 questionnaire concept [9, 13], a different technique was proposed to compute COVID-19 HL levels based on Rasch-Thurstonian thresholds [14, 15]. The advantage of this technique is that people are classified into different levels based on their person measure and the probabilities of choosing a response category rather than adopting cut-off values from other questionnaires, respectively, on arbitrary cut-off values. Using Rasch-Thurstonian thresholds, 0% of school principals were categorized into Level 1, 3.4% into Level 2, 68.6% into Level 3 and 28% into Level 4 of self-reported COVID-19 HL. Nevertheless, a theory-based classification system that divides people into different competency levels on item-level would be best, dividing people by having reacted to specific items, operationalizing a specific competence, differently (e.g. choosing “right vs. wrong” or “easy vs. difficult”) [14].

### Regression analysis

More important than classifying people into HL levels is if COVID-19 HL can predict health-related outcomes. Multiple linear regression analysis was conducted using COVID-19 HL person measures and revealed that COVID-19 HL was a relevant factor associated with COVID-19 related HPS implementation for all three subscales (students, staff, school). This is in line with previous findings that general HL and COVID-19 HL positively predicted HPS implementation [43, 65, 66]. In addition to that, school principals’ COVID-19 HL was found to be associated with their own health-related outcomes in other countries that conducted the COVID-HL school survey [58, 67]. School principals with low COVID-19 HL were more likely to report psychosomatic complaints and exhaustion related to work situation in the Hong Kong COVID-HL school survey [58]. In the Taiwan COVID-HL school survey, school principals with higher COVID-19 HL were less likely to report perceived stress, depressive symptoms and COVID-19 related fear [67]. These findings underline the proposed potential of HL as a personal resource for school principals, but also as a resource on a school level as school principals can be seen as gatekeepers for school health promotion and prevention [28]. School principals’ health literacy and associations with health-related outcomes need to be further investigated in future studies.

Next to COVID-19 HL, sociodemographic characteristics of school principals were included as explanatory variables in the regression models. Overall, female school principals, older school principals and school principals working at a primary school tended to report higher COVID-19 related HPS implementation. This is in line with a pre-pandemic German study that revealed significant associations between low HPS implementation and male gender, younger age and working at a secondary school [44]. In the Swiss COVID-HL school survey, older school principals tended to report higher levels of COVID-19 related HPS implementation, while no sex differences were found [65]. However, in the Swiss study, the analyses were conducted for the whole COVID-19 related HPS implementation scale and not differentiated by the three subscales. Since sociodemographic differences were not found for every subscale in the present study, it would be highly interesting to investigate differences in relation to the different dimensions (gender, age, school type) of a holistic approach to HPS in future studies. The current results highlight the need to shed a light on which characteristics of school principals might influence HPS implementation. Especially health indicators, since it has been suggested that school principals’ health might be relevant for HPS implementation [65, 68].

### Limitations

Due to the cross-sectional study design, the results should be interpreted cautiously and no causal relationships can be drawn. The survey has been conducted in only four out of the 16 German federal states. Due to missing public statistics, no data are available on the basic population of school principals or their gender ratio. It is therefore not representative for all school principals, school principal deputies and members of the school management team in Germany. Furthermore, the current sample is particular and homogenous (mainly female school principals from primary schools at the age of around 51 years) and cannot be accounted for a general adult population. Additionally, a self-report bias cannot be excluded. The variance explained in the regression models of the COVID-19 related HPS implementation subscales was relatively low. Indeed, other factors influence HPS implementation and need to be investigated in future studies. The purpose of the present study was to investigate the association between self-reported COVID-19 HL and COVID-19 related HPS implementation.

## Conclusions

School principals’ self-reported COVID-19 HL seemed to be a relevant factor associated with the implementation of health promotion in schools during the pandemic. Alongside investing in school principals’ health and well-being, particular emphasis should be directed toward enhancing their health literacy. From a settings-based perspective aiming at improving schoolchildren’s health and HL, strengthening school principals’ HL can be considered a structural intervention to modify children’s and adolescent’s environment and should be integral to HPS approaches. The proposed Rasch-Thurstonian threshold technique might be better suited to classify people into different levels than previous classifications for current self-report HL questionnaires. However, if people need to be classified into competency levels, the classification system should be based on theoretical competence differences, operationalized in a questionnaire on item-level. Finally, the sociodemographic indicators gender, age, and school type seem to influence the implementation rate of school health promotion and should be investigated further.

## Supplementary Information

Supplementary Material 1. Supplementary Table 1. Items of the COVID-19 related HPS implementation scale. Table in which the item descriptions of the COVID-19 related HPS implementation scale are displayed. This table was requested by Reviewer 1 and helps to understand the content of the questionnaire.

Supplementary Material 2. Supplementary Table 2. Estimated person measures for all possible raw scores. Table in which the estimated person measures, computed by the Rasch analysis, are shown for every possible raw score that a person could have achieved on the HLS-COVID-Q22 questionnaire. This table helps to understand the computation of the COVID-19 HL levels.

## Data Availability

The dataset generated and analyzed during the current study is not publicly available as data analyses has not been finished. When data analyses are completed, the dataset will be made publicly available. Until then, the data are available from the corresponding author upon reasonable request.

## Abbreviations

COVID-HL Network: COVID-19 Health Literacy Network
COVID-HL school survey: COVID-19 Health Literacy School Principals Survey
GLOBHL: Global Health Literacy Research Network
HL: Health literacy
HPS: Health promoting school
VIF: Variance inflation factor
