# Supplementary material for: Association of school leaders’ COVID-19 health literacy with the implementation of health promotion in schools in Germany: a cross-sectional study
Source: BMC Public Health. 2025 Aug 21;25:2875. doi: 10.1186/s12889-025-24196-9 (PMC12369071; doi:10.1186/s12889-025-24196-9)
Supplement: Supplementary file 2 — Supplementary Material 2. Supplementary Table 2. Estimated person measures for all possible raw scores. Table in which the estimated person measures, computed by the Rasch analysis, are shown for every possible raw score that a person could have achieved on the HLS-COVID-Q22 questionnaire. This table helps to understand the computation of the COVID-19 HL levels. [file 12889_2025_24196_MOESM2_ESM.docx]

Supplementary Table 2. Estimated person measures for all possible raw scores.

| **Raw score** | **Person measure** | **SE** | **Raw score** | **Person measure** | **SE** |
| --- | --- | --- | --- | --- | --- |
| 22 | -7.36E | 1.84 | 56 | -.10 | .37 |
| 23 | -6.12 | 1.03 | 57 | .04 | .38 |
| 24 | -5.37 | .75 | 58 | .18 | .38 |
| 25 | -4.91 | .62 | 59 | .33 | .39 |
| 26 | -4.56 | .55 | 60 | .48 | .39 |
| 27 | -4.28 | .51 | 61 | .64 | .39 |
| 28 | -4.04 | .48 | 62 | .79 | .40 |
| 29 | -3.83 | .45 | 63 | .95 | .40 |
| 30 | -3.63 | .43 | 64 | 1.12 | .40 |
| 31 | -3.45 | .42 | 65 | 1.28 | .40 |
| 32 | -3.28 | .40 | 66 | 1.44 | .40 |
| 33 | -3.12 | .39 | 67 | 1.61 | .41 |
| 34 | -2.97 | .39 | 68 | 1.77 | .41 |
| 35 | -2.82 | .38 | 69 | 1.93 | .41 |
| 36 | -2.68 | .37 | 70 | 2.10 | .41 |
| 37 | -2.55 | .37 | 71 | 2.27 | .41 |
| 38 | -2.41 | .36 | 72 | 2.43 | .41 |
| 39 | -2.28 | .36 | 73 | 2.60 | .41 |
| 40 | -2.15 | .36 | 74 | 2.77 | .42 |
| 41 | -2.02 | .36 | 75 | 2.95 | .42 |
| 42 | -1.89 | .35 | 76 | 3.13 | .43 |
| 43 | -1.77 | .35 | 77 | 3.31 | .43 |
| 44 | -1.65 | .35 | 78 | 3.50 | .44 |
| 45 | -1.52 | .35 | 79 | 3.70 | .45 |
| 46 | -1.40 | .35 | 80 | 3.92 | .47 |
| 47 | -1.27 | .35 | 81 | 4.15 | .49 |
| 48 | -1.15 | .35 | 82 | 4.40 | .51 |
| 49 | -1.02 | .35 | 83 | 4.68 | .55 |
| 50 | -.90 | .36 | 84 | 5.00 | .59 |
| 51 | -.77 | .36 | 85 | 5.38 | .66 |
| 52 | -.64 | .36 | 86 | 5.89 | .77 |
| 53 | -.51 | .36 | 87 | 6.68 | 1.05 |
| 54 | -.38 | .37 | 88 | 7.95E | 1.85 |
| 55 | -.24 | .37 |  |  |  |

Note. SE = standard error.
