# Supplementary material for: Association of school leaders’ COVID-19 health literacy with the implementation of health promotion in schools in Germany: a cross-sectional study
Source: BMC Public Health. 2025 Aug 21;25:2875. doi: 10.1186/s12889-025-24196-9 (PMC12369071; doi:10.1186/s12889-025-24196-9)
Supplement: Supplementary file 1 — Supplementary Material 1. Supplementary Table 1. Items of the COVID-19 related HPS implementation scale. Table in which the item descriptions of the COVID-19 related HPS implementation scale are displayed. This table was requested by Reviewer 1 and helps to understand the content of the questionnaire. [file 12889_2025_24196_MOESM1_ESM.docx]

Supplementary Table 1. Items of the COVID-19 related HPS implementation scale.

| **Item** | **Now we have a few questions about whether and to what extent health issues are addressed at your school in the context of the current COVID-19 pandemic, among other things. At our school, …** |
| --- | --- |
| 1 | …students are taught basic information about the coronavirus (e.g. causes of its  development, spread). |
| 2 | …students learn ways to protect themselves from infection. |
| 3 | …students learn how to get enough exercise despite the restrictions due to the coronavirus. |
| 4 | …students learn how to eat healthily despite the restrictions due to the coronavirus. |
| 5 | …students are supported in dealing with worries and fears caused by the coronavirus. |
| 6 | …school staff are supported in dealing with stressful situations caused by the coronavirus (e.g. stress). |
| 7 | …health-promoting aspects play an important role in the design of teaching and learning conditions (including homeschooling). |
| 8 | …health-promoting aspects play an important role in the design of working conditions (including home office). |
| 9 | …there are regular further training courses on health-related topics (protection against infection with the coronavirus, dealing with stressed students). |
| 10 | …stress resulting from the COVID-19 pandemic (workloads, student stress) are regularly addressed. |
| 11 | …we work closely with parents when it comes to promoting and protecting children's health. |
| 12 | …we work closely with community stakeholders from the health and social sectors when it comes to promoting and protecting the health of our students. |
| 13 | …there is a consensus that health and school performance of students are interrelated. |
| 14 | …students are involved in the planning of prevention and health promotion activities. |
| 15 | …(digital) spaces of social interaction and exchange are created despite the corona-related restrictions. |

Note. The German version of the COVID-19 related HPS implementation scale was used in the study. For reporting purposes, the English version of the items is displayed. 3 subscales: HPS-students (Items 1-5), HPS-staff (Items 6-8), HPS-school (Items 11-14). Items 9, 10 and 15 items were excluded for data analysis. Response options: [1] Not true at all, [2] Mostly not true, [3] Likely to be true, [4] Totally true. Reference: Dadaczynski K, Okan O, Messer M. COVID-19 Health Literacy School Principals Survey (COVID-HL: School Principal): Questionnaire and Scale Documentation. Public Health Centre Fulda (PHZF) at Fulda University of Applied Sciences & Interdisciplinary Centre for Health Literacy Research (IZGK) at Bielefeld University. 2021: doi: 10.4119/unibi/2952552.
